# Fracture Toughness, Compressive Strength and Fracture Surface Morphology of Cement Mortars Modified with Nano-SiO_2_

**DOI:** 10.3390/ma18245516

**Published:** 2025-12-08

**Authors:** Wioleta Iskra-Kozak, Janusz Konkol, Marek Poręba

**Affiliations:** 1 Faculty of Civil and Environmental Engineering and Architecture, Rzeszow University of Technology, Poznanska 2, 35084 Rzeszow, Poland; janusz.konkol@prz.edu.pl; 2 The Faculty of Mechanical Engineering and Aeronautics, Rzeszow University of Technology, Al. Powstańców Warszawy 8, 35084 Rzeszow, Poland; poreba@prz.edu.pl

**Keywords:** cement composites, nano-silica, fracture toughness, compressive strength, fractal dimension

## Abstract

**Highlights:**

**What are the main findings?**

- Nano-silica improves fracture toughness and strength of cement composites.
- Strong link found between fracture surface morphology and mechanical properties.

**What are the implications of the main findings?**

- Lower water/binder ratio and higher nano-silica content enhance the overall performance of the material.
- Predictive models can be used to estimate fracture toughness and compressive strength.

**Abstract:**

This study investigates the effect of nano-SiO_2_ on the mechanical properties and fracture surface morphology of cement composites. The focus was on fracture toughness (*K_Ic_^S^*) and compressive strength. Composites with nano-SiO_2_ showed up to 63% higher fracture toughness and 68% higher compressive strength than unmodified ones. The influence of nano-SiO_2_ content (0.09–2.91% by binder mass) and water-to-binder ratio (0.43–0.57) was examined using a Central Composite Design. Properties improved with higher nano-SiO_2_ content and lower *w/b* ratio. Fractographic analysis using laser profilometry revealed a clear correlation between the fractal dimension (*D*) of fracture surfaces and *K_Ic_^S^* and fcm. Lower *D* values indicated smoother fracture surfaces and denser microstructures. The results clearly show that changes in the composition—particularly the nano-SiO_2_ content and the water-to-binder ratio—determine the development of the microstructure, which in turn governs the mechanical properties of the material. Consequently, the study establishes a coherent, quantitative relationship between mixture composition, fracture microstructure, and the macroscopic properties of the composite.

## 1. Introduction

Modern engineering of building materials, particularly those with a cement matrix, places increasing demands not only on the introduction of new modifiers for concrete composition but primarily on the analysis of the impact of these modifications on the microstructure, properties, as well as the durability and usability of this material. One of the promising modifiers of cement composites is, among others, nanosilica (nano-SiO_2_). In an era when the cement industry is seeking alternatives to energy-intensive cement production processes that are associated with high CO_2_ emissions, as well as potential substitutes for traditionally used silica fume, research into nanomaterials is becoming particularly significant.

In recent years, nanomaterials have become one of the most promising groups of additives used in cement composites due to their unique physicochemical properties and their ability to modify the material’s microstructure at the nanometric scale. Studies on nanomaterials used in cement composites show that their effectiveness in modifying the microstructure is closely linked to maintaining stable dispersion, as confirmed by Chen et al. [1]. At the same time, Gao et al. [2] indicate that the high pH of the cementitious environment promotes the re-agglomeration of nanoparticles, which significantly limits the efficiency of nanomodification. In recent years, nano-SiO_2_ has become one of the most extensively studied nanoadmixtures used in cement composite technology, primarily due to its ability to modify the microstructure at the nanometric scale and to enhance the durability and mechanical performance of the material. The latest literature reviews [3] clearly indicate that nanosilica, owing to its very high specific surface area and strong pozzolanic reactivity, significantly influences hydration processes by increasing the amount of C-S-H phase, reducing portlandite, and densifying the cement paste structure. The authors also emphasize the key role of nano-SiO_2_ in densifying and strengthening the interfacial transition zone (ITZ), which leads to improvements in both mechanical strength and resistance to environmental degradation. The presence of nanosilica in cementitious composites does not merely serve as a physical micro-filler. The introduction of nanosilica primarily enhances the structural properties of cement composites. Nanosilica particles exhibit the ability to absorb calcium ions. Moreover, they act as nucleation sites, causing a significant acceleration of cement phase hydration. As a result of the reaction between nanosilica and cement grains, H_2_SiO_4_^2−^ ions are formed. These ions react with available Ca^2+^, producing an additional amount of C-S-H phase. The formation of C-S-H does not occur solely at the surface of the cement grains, as in pure C_3_S hydration, but also within the pore solution. The generation of a large number of nucleation sites accelerates the early hydration of cement [4]. The hydration of Portland cement is an exothermic process. It has been demonstrated that the hydration process of cement modified with nano-SiO_2_ follows a similar pattern to that of unmodified Portland cement [5,6]. Furthermore, increasing the nano-SiO_2_ content leads to an acceleration of hydration. The higher the dosage of nano-SiO_2_, the higher the rate of heat release during hydration [7].

The research conducted by Hu and Li [8,9] offers foundational insights into this area, demonstrating that the integration of nano-SiO_2_ and nano-Fe_2_O_3_ markedly enhances the compressive and flexural strength of cement mortars. Similar results have been confirmed by other researchers, who highlighted the advantages of nano-SiO_2_ [10,11,12,13,14,15,16,17].These studies suggest that nano-SiO_2_ not only improves mechanical strength but also accelerates the initial strength of cement composites by promoting cement hydration and facilitating the pozzolanic reaction. Despite the numerous benefits associated with nano-SiO_2_, comparable effects in concrete technology have not been observed to the same extent as with silica fume. The existing literature on the fracture toughness of nanosilica-modified concretes is limited. Research indicates that fracture energy increases with colloidal nanosilica content up to 3%, after which it decreases with higher concentrations in semi-lightweight self-compacting concrete (SCSL) [18]. It was observed that concrete with a lower water/binder ratio exhibited greater fracture energy, reinforcing the relationship between fracture energy and compressive strength. The critical stress intensity factor increased by 24% when the water-to-solid ratio decreased from 0.45 to 0.35 [18]. In contrast, Zhang et al. [19] reported that fracture parameters improved with increased nanosilica content up to 5%, followed by a decline with additional nano-SiO_2_ in fly ash concrete composites. Zhang et al. [20,21] conducted a series of three-point bending tests to assess the effect of SiO_2_ nanoparticles on the fracture parameters of high-performance concrete (HPC) containing 15% fly ash. Their findings revealed that HPC modified with nanosilica exhibited superior fracture parameters compared to that without SiO_2_ nanoparticles. The tests showed that when the nanoparticle content remained below 5% relative to the binder mass, fracture parameters gradually improved with higher SiO_2_ nanoparticle content; however, exceeding 5% resulted in a slowdown of this trend [20,21].

One of the primary motivations for researchers investigating cement composites to focus on fractal geometry is the pursuit of a more precise understanding and characterization of the fracture mechanisms inherent to these materials. Winslow [22] demonstrated the applicability of fractal geometry in characterizing cement composites in 1985, using X-ray techniques to reveal the fractal nature of hydrated cement paste surfaces. Notably, Winslow observed that the fractal dimension of these surfaces diminished as the water-cement ratio (*w/b*) increased, indicating a correlation between the material’s composition—which significantly influences its microstructure—and the fractal dimension that reflects the roughness of this microstructure. Building upon findings from steel research, subsequent investigations sought to establish a relationship between the fractal dimension of fracture surfaces and fracture mechanics parameters. Saouma and Barton [23] were among the first to study this connection, noting that an increase in the fractal dimension corresponded with a decrease in fracture energy (*G_F_*) and the critical stress intensity factor (*K_Ic_^S^*), with the derived relationships appearing linear. Yan et al. [24] and Issa et al. [25] confirmed this linear relationship between fractal dimension and fracture energy in concretes with varying water/binder ratios, while Prokopski and Langier [26] demonstrated similar correlations pertaining to the critical stress intensity factor (*K_Ic_^S^*) as a measure of fracture resistance. Further studies by Prokopski and Konkol [27] examined concretes based on three distinct aggregate types: basalt, gravel, and dolomite cured for intervals of 7, 14, 28, and 90 days. They established linear relationships between the critical stress intensity factor (*K_Ic_^S^*), modulus of elasticity in bending (*E*), compressive strength (*f_cm_*), and the fractal dimension (*D_c_*), which was assessed using the chord method. Their observations indicated that an increase in the fractal dimension coincided with improvements in all tested mechanical parameters. Additionally, it was noted that as concrete aged, the values of all parameters (*K_Ic_^S^*, *f_cm_*, *E*) increased, while the fractal dimension for the same concrete remained constant. Notably, high-strength concretes exhibited lower values of fractal dimension *D* compared to ordinary concretes, which is attributed to the reduced roughness of the fracture surfaces in high-strength concretes.

The demonstrated correlation between the properties of cementitious composites and the fracture surface formed as a result of material failure has prompted researchers to seek models capable of simply describing the complex phenomenon of concrete fracturing. The application of fractal geometry to address this issue enables the derivation of an infinite number of potential crack propagation paths in concrete as final solutions, each described by curves characterized by an invariant fractal dimension that is, constant roughness. This approach, which employs so-called deterministic chaos, was presented by Konkol in his study [28]. He proposed the shape of a generator, along with the method for its construction and simultaneous simulation of the fracturing phenomenon. This method is universal and can be successfully applied to simulation processes involving other materials as well.

Despite the extensive body of research on nanosilica, the literature still lacks studies that quantitatively link the morphology of the fracture surface—described by the fractal dimension determined using laser profilometry—with the fracture resistance of cement composites modified with nano-SiO_2_. The aim of the present study is to evaluate the effect of varying nanosilica content, used as a modifier in cementitious composites with different water-to-binder ratios, on the mechanical parameters of these composites, including fracture toughness, as well as on their microstructure, reflected by the roughness of the fracture surface formed during material failure. Attempts to develop predictive models that incorporate mortar age, nano-SiO_2_ content, and microstructural parameters in the context of mechanical performance are also rarely reported. The predictive models developed in this work enable the estimation of fracture toughness based on the age of the mortar and allow correlating fracture resistance with microstructural features characterized using fractographic methods. This approach can be effectively applied in the design of cementitious composites with targeted properties. Establishing these relationships makes it possible to tailor the microstructure of nanosilica-modified mortars through the appropriate selection of key formulation variables, such as the water-to-binder ratio and nano-SiO_2_ content, in order to achieve desired material characteristics. Moreover, correlations were demonstrated between the fractal dimension *D* of the profile line extracted from the fracture surface of cementitious composites and their fracture toughness. Therefore, the novelty of this study lies in quantitatively linking composition, microstructure, and macroscopic mechanical properties, as well as in developing regression models that enable predicting fracture resistance based on nano-SiO_2_ content, water-to-binder ratio, and the fractal dimension of the fracture surface.

## 2. Materials and Methods

### 2.1. Materials Used

Portland cement CEM I 42.5 N, produced by Cement Ożarów S.A (Ożarów, Poland). and compliant with the PN-EN 197-1 standard [29], was used in the study. The chemical and mineralogical composition of the Portland cement is presented in Table 1. The chemical and mineralogical composition of the cement was provided by the manufacturer (Cement Ożarów S.A.) based on the official technical specification of the material.

Tap water meeting the requirements of PN-EN 1008:2004 [30] was used in the cementitious composite mixtures.

Nanosilica (nano-SiO_2_) with a particle size up to 15 nm, produced by Alroko GmbH & Co KG (Hamburg, Germany), was used as a modifier in the cementitious composites. The applied nanosilica was obtained through physical vapor deposition (PVD) from the gas phase. The properties of the nanosilica used are presented in Table 2.

**Figure 1:**
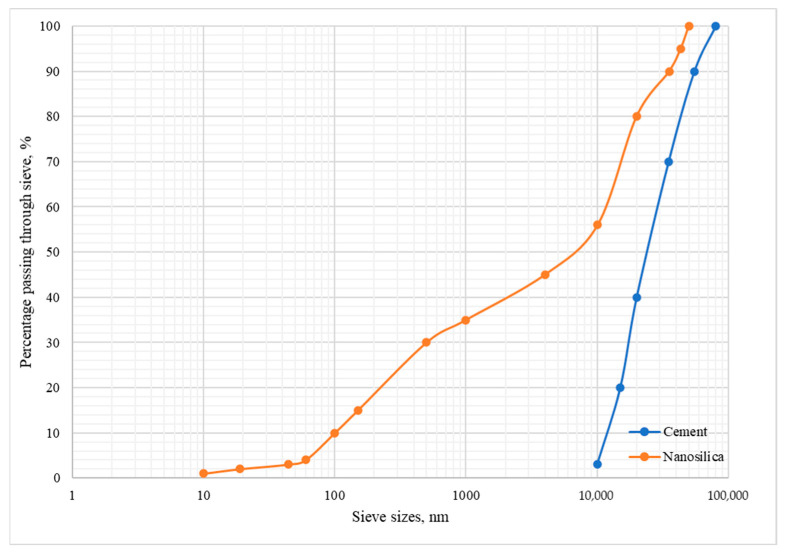
Particle size distribution of cement and nanosilica.

The NanoPlus HD wet dispersion system, capable of measuring nanoparticles ranging from 0.1 nm to 12,300 nm, was used to determine nanoparticle size. Ethanol was used as the dispersing liquid. A similar method was applied to analyze the particle size distribution of the cement. Both analyses were performed at the Department of Materials Science, Faculty of Mechanical Engineering and Aeronautics, Rzeszów University of Technology. The results are presented graphically in Figure 1.

The particle size distribution of cement and nanosilica (Figure 1) differs significantly, which directly affects their properties and roles in cement composites. Cement particles fall within the range of 10,000 to 80,000 nm, whereas nanosilica is characterized by a much finer grain size, with particles ranging from approximately 10 to 50,000 nm. The smaller size of nanosilica particles results in a greater specific surface area, which in turn leads to high reactivity and the ability to modify the properties of cement, including the improvement of the microstructure and strength of the composites. However, it is worth noting that the obtained results of the nanosilica particle size distribution analysis did not confirm the parameters declared by the manufacturer, which limited the effectiveness of this composition modifier. The most probable cause of this discrepancy is the strong tendency of nano-SiO_2_ to form agglomerates, resulting from its very high specific surface area and high surface energy. Ultrasonic sonication was applied to improve the dispersion of nano-SiO_2_ in the mixing water; however, complete breakdown of all agglomerates could not be achieved. It should be emphasized that despite agglomeration, nano-SiO_2_ retains high pozzolanic activity.

Quartz sand with a grain size of up to 2 mm and a bulk density of 2.65 kg/dm^3^ from the “ZEK Lipie KruszGeo” quarry was used as the fine aggregate. The sand meets the requirements of the PN-EN 12620+A1:2010 [31] standard.

### 2.2. Experimental Methods

**Figure 2:**
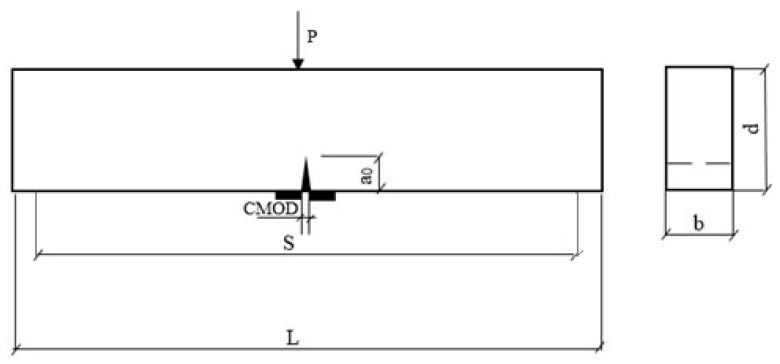
Diagram of the specimen used in tests according to the Mode I fracture model [34]:; *CMOD*—crack mouth opening displacement; *S*—span between supports, m; *a_0_*—initial notch height, m; *d*, *b*—specimen height and width, m; *L*—specimen length, m.

The scope of experimental methods included conducting the following tests:

- Sample preparation. Mixtures of unmodified and nanosilica-modified cement composites were prepared using specified proportions of components: cement, standard sand, water, and a modifier in the form of nano-SiO_2_. The mixing process was carried out in a laboratory mixer meeting standard requirements. The mixtures were placed in molds in two layers, each of which was compacted mechanically on a vibrating table (60 vibrations within 60 s). After compaction, the molds were covered with foil and stored for 24 h under laboratory conditions (temperature 20 ± 2 °C, relative humidity approximately 65%). After this period, the samples were demolded and inspected for possible damage, then transferred to a climate chamber. The samples were stored above water (temperature 20 ± 2 °C, humidity above 95%) in a horizontal position until the time of testing.
- The consistency of the fresh mortar was tested using a flow table in accordance with PN-EN 1015-3:2000/A2:2007 [32]. The measure of mortar consistency was the flow value, determined by measuring the average diameter of a sample of fresh mortar subjected to vertical shocks through the lifting and free dropping of the table’s plate from a specified height (15 shocks within 15 s).
- The compressive strength of the mortars was tested after 28 and 90 days of curing in accordance with PN-EN 1015-11:2001 [33]. The compressive strength was determined using the halves of three prism specimens that remained after the mortar flexural strength test.
- Fracture resistance tests of the mortars according to mode-I fracture toughness were conducted in accordance with the draft recommendations of RILEM [34] using a strength testing machine capable of applying a constant displacement increment. Due to the maximum aggregate size of up to 16 mm, the tests were carried out on beams with dimensions of 40 mm × 80 mm × 360 mm, with an initial notch *a*_0_ of a length equal to 1/3 of the beam’s height, made during the sample molding process. On each sample, blades for mounting the extensometer were placed on both sides of the initial notch *a*_0_. The specimen layout is shown in Figure 2. During the test, the relationship between force and crack mouth opening displacement was recorded continuously.
- Analysis of the morphology of fracture surfaces of mortar beams damaged during testing was conducted using a Talysurf CLI 1000 laser profilometer by Taylor Hobson (Warrenvile, USA), together with dedicated software and the FRAKTAL_Wymiar2D program (J. Konkol, 2000, Poland) [28]. On each surface, 20 profile lines of 30 mm in length were marked. The required number of profile lines was determined based on analyses described in [28]. Measurements were carried out with a discretization step of 1 µm, resulting in 30,001 points describing each profile line. The fractal dimension *D* was determined using the box-counting method. The box-counting method (*D_BC_*) is a variant of the covering method in which the analyzed structure is covered with boxes. This method is universal and can be used for any structures, including 3D ones [28]. It involves overlaying a grid with a known side length on the analyzed profile line and then counting the number of boxes containing the structure. The fractal dimension is calculated based on the slope of the line representing the logarithmic relationship between the number of boxes and the inverse of the box size.

## 3. Results and Discussions

### 3.1. Preparation of the Mixture, Nanoparticle Dispersion

The experimental design was generated using the Statistica (Version 13.3.) software. A central composite design was selected, which required conducting experiments at 9 design points with repetitions at all points. The composition of the composites was described using two independent variables: the water to binder (*w/b*) ratio (binder = cement + nanosilica) and the percentage of nanosilica relative to the binder mass (*NS/b*). The water-to-binder ratio ranged from 0.429 to 0.57, while the nanosilica content ranged from 0.086% to 2.914% of the binder mass. The precision of the independent variables was determined by the adopted experimental design.

In addition to the composites modified with nano-SiO_2_, control composites without nanosilica were also prepared, with water-to-cement ratios of 0.45 (series A), 0.5 (series B), and 0.55 (series C).

The quantities of components for modified and unmodified composites per 1 m^3^ are summarized in Table 3.

An important stage in the preparation of the mortars was the development of a method for introducing nanoparticles into the mix. The use of a sonicator was considered appropriate for this purpose. The study by Gao et al. [35] highlights that achieving stable dispersion of nanomodifiers within the cement matrix is one of the main challenges, as the high-pH environment and the presence of Ca^2+^ ions promote the re-agglomeration of nanoparticles even after they have been initially dispersed using ultrasonication. A constant ultrasonic sonication time was adopted as a preliminary assumption. The study used a Sonics VC505 ultrasonic disintegrator with a nominal power of 200 W and a vibration frequency of 20 kHz. A titanium sonotrode with a diameter of 13 mm and a vibration amplitude of 170 µm (70%) was applied. This process is crucial for achieving the desired effects in composite modification. The research was conducted at the Department of Environmental Engineering and Chemistry, Faculty of Civil and Environmental Engineering and Architecture, Rzeszów University of Technology. In the initial phase of the study, SiO_2_ nanoparticles in powder form were mixed with a fixed amount of mixing water (180 mL) and subjected to ultrasonic sonication for 10 min, regardless of the quantity of nano-SiO_2_ added. The prepared mixture was then combined with the pre-mixed dry mortar components. Additionally, a polycarboxylate-based superplasticizer, MasterEase 3040, was introduced to maintain the proper workability of the resulting mixtures.

However, significant variability was observed in the compressive strength results of mortars after 3 and 7 days of curing. Considerable differences in the dispersion measures of compressive strength were particularly noticeable in mortar series with the highest nano-SiO_2_ content of 2.9% relative to the binder mass. Preliminary compressive strength test results after 3 and 7 days of curing for three samples with a *w/b* ratio of 0.5 and a nano-SiO_2_ content of 2.9%, as well as with a *w/b* ratio of 0.55 and a nano-SiO_2_ content of 0.5% relative to the binder mass, are presented in Table 4.

Based on further research, a technological solution was ultimately proposed involving a variable ultrasonic sonication time, depending on the nanosilica content. An improvement in the compressive strength after 3 and 7 days of curing was observed in nano-SiO_2_ modified cementitious composites, which helped reduce the previously noted significant variability within individual mortar series. SiO_2_ nanoparticles in powder form were again mixed with 180 mL of mixing water and subjected to ultrasonic sonication. The sonication time was determined experimentally, depending on the amount of nanosilica introduced into the mortar mix. The duration of ultrasonic treatment was increased proportionally to the nanosilica content. The resulting mixture was added to the dry mortar components immediately after ultrasonic treatment. A polycarboxylate-based superplasticizer, MasterEase 3040, was once again used to maintain consistent workability of the resulting mixes. Table 5 (samples for compressive strength testing) and Table 6 (samples for Mode I fracture toughness testing) present the relationship between ultrasonic sonication time and the amount of nano-SiO_2_ introduced. Differences in sonication times are due to the varying sizes of the samples: those intended for fracture toughness testing according to Model I are larger than those for compressive strength testing, necessitating adjustments to the process parameters.

**Figure 3:**
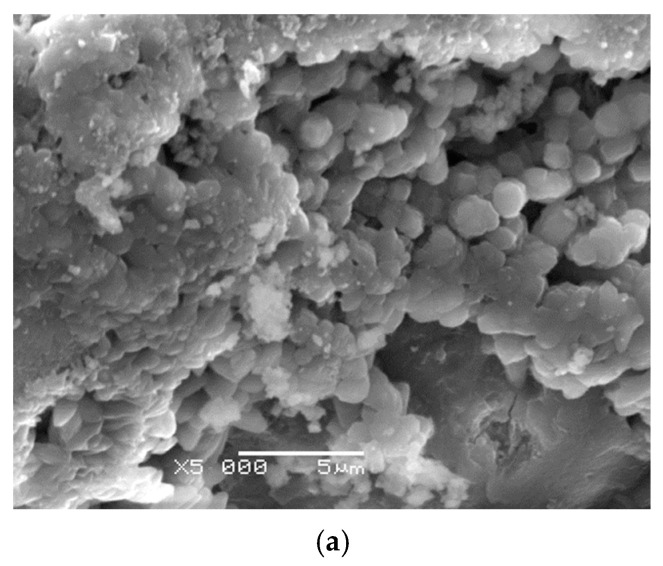

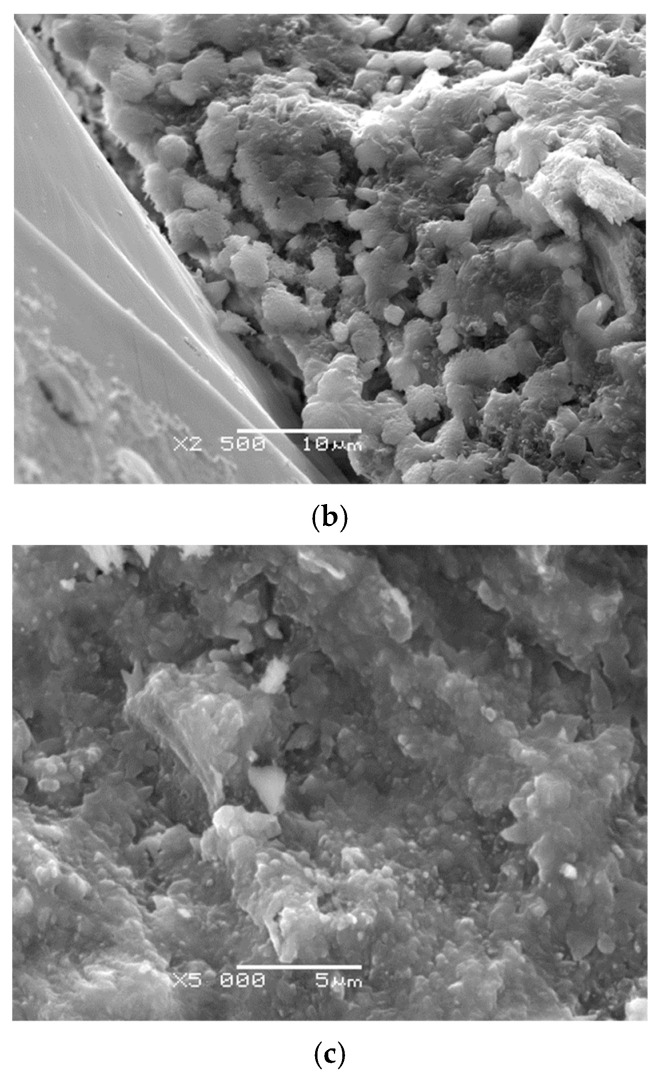
(**a**–**c**) SEM microstructure of mortar: reference series A (*w*/*c* = 0.45) (**a**), series 9 with 1.5% nano-SiO_2_ (*w*/*c* = 0.50) (**b**) and series 1 with 2.5% nano-SiO_2_ (*w*/*c* = 0.45) (**c**) after 180 days of curing.

The effectiveness of the applied sonication process is confirmed by the microstructural analysis of the mortars shown in the SEM images. In the reference mortar, without nanosilica, a distinctly more porous and heterogeneous structure of the hardened cement paste is observed, along with a less developed interfacial transition zone (ITZ) (Figure 3a).

In contrast, the mortar modified with 1.5% nano-SiO_2_ exhibits a clearly densified microstructure, reduced voids, and a greater amount of hydration products (Figure 3b). A similar effect is observed in the mortar containing 2.5% nano-SiO_2_, where the microstructure is even more compact, indicating enhanced filling of capillary pores and a more uniform ITZ (Figure 3c). These observations confirm that the appropriately selected sonication time enabled effective dispersion of nanosilica, resulting in improved microstructure and enhanced mechanical properties of the composite.

### 3.2. Consistency

The consistency of fresh mortar modified with nanosilica, introduced as a partial replacement for cement, is significantly influenced by the amount of this modifier. Due to the high surface area to volume ratio of its particles, nano-SiO_2_ exhibits high water demand. Numerous rheological studies on nano-SiO_2_ modified mortars have demonstrated the absolute necessity of using superplasticizers to achieve high workability and to reduce excessive air void content [5].

To meet the requirement of maintaining a consistent workability across all mortar mixes, an experimentally determined amount of the polycarboxylate-based superplasticizer MasterEase 3040 was added. The amount of water introduced into the mortar mix was adjusted to account for the water content provided by the superplasticizer. Workability was assessed using the flow table method, with a target flow diameter of 160 mm ± 10 mm. All tested mixes exhibited high workability and good consistency stability. The results of the flow table tests for cementitious composites without nano-SiO_2_ and those modified with nano-SiO_2_ are presented in Table 7.

**Figure 4:**
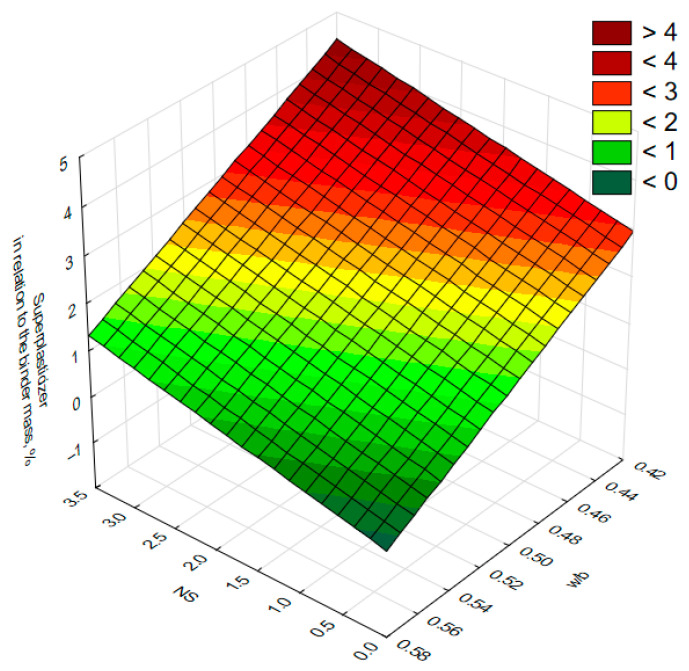
Superplasticizer demand as a function of the water to binder ratio (*w/b*) and nano-SiO_2_ content (*NS*) relative to binder mass.

Due to its high surface area-to-volume ratio, nanosilica exhibits a significant capacity for water absorption. The incorporation of nano-SiO_2_ into cement mortars led to a deterioration in the consistency of the mortar mixes as the amount of nanosilica increased. An analysis of the quantity of superplasticizer used (Figure 4) revealed that the demand for superplasticizer rises with decreasing water to binder ratio and increasing nanosilica content. This relationship was described using a regression model that accounts for the influence of both the water to binder ratio and the nanosilica content on the amount of superplasticizer required. The resulting equation is presented below:

SP=11.1−19.4·w/b+41.7·NS

where *SP*—percentage of superplasticizer relative to binder mass; *w/b*—water to binder ratio; *NS*—percentage of nanosilica relative to binder mass

The results obtained in the present study are consistent with the findings of other researchers, who also emphasize the strong influence of nanosilica on the rheological and microstructural properties of cement mortars. Due to its very high specific surface area relative to particle volume, nano-SiO_2_ exhibits high water demand, which leads to a reduction in the amount of free water in the mixture as its content increases. This phenomenon has been widely reported in the literature, where the necessity of using superplasticizers to maintain adequate workability and to limit excessive air entrainment is highlighted. The consistency between the trends observed in our investigation and those documented by other authors confirms the validity of the adopted experimental assumptions and the reliability of the obtained results [9,10].

### 3.3. Compressive Strength and Fracture Toughness Tests

Based on the obtained results of compressive strength (*f_cm_*) and fracture toughness expressed by the critical stress intensity factor (*K_Ic_^S^*) of nanosilica-modified cementitious composites after 28 and 90 days of curing, an increase was observed across all tested series. The test results are presented in Table 8.

The highest compressive strength after 28 days of curing was obtained for mortar from series 1 (*w/b* = 0.45, 2.5% *NS*), while after 90 days of curing, the highest compressive strength was achieved by mortar from series 7 (*w/b* = 0.5, 2.9% *NS*). After 28 days, series 1 mortar reached 60 MPa, representing a 40% increase compared to unmodified mortars with the same water to binder ratio, and after 90 days the increase was 30%. The highest observed increase in compressive strength was 55% after 28 days and 41% after 90 days of curing, recorded for mortar from series 7.

The highest value of the critical stress intensity factor (*K_Ic_^S^*) after 28 and 90 days of curing was obtained for the mortar from series 7 (*w/b* = 0.5, 2.9% *NS*). This mortar exhibited the greatest increase in *K_Ic_^S^* compared to unmodified mortars. After 28 days of curing, *K_Ic_^S^* increased by 47%, and after 90 days, a 63% increase was observed relative to the unmodified mortar with the same water to binder ratio (*w/c* = *w/b*). As the *w/b* ratio decreases, the *K_Ic_^S^* value increases, regardless of curing time.

**Figure 5:**
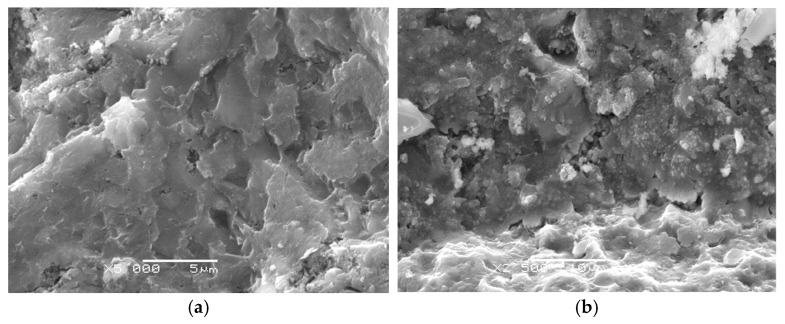
(**a**,**b**) Microstructure of series 7 mortar modified with 2.914% nano-silica at *w/b* = 0.5 after 28 days of curing.

The conducted research revealed that even a small amount of nano-SiO_2_ relative to the binder mass leads to a significant increase in compressive strength and crack resistance, expressed by the critical stress intensity factor (*K_Ic_^S^*), compared to mortars unmodified with nanosilica and with the same water to binder (*w/b*) ratio. As the content of nano-SiO_2_ increases and the *w/b* ratio decreases, further improvement in compressive strength and fracture toughness of the mortars is observed. The enhancement of the mechanical properties of mortars modified with nanosilica is primarily influenced by the cement hydration process and the high pozzolanic activity of nano-SiO_2_. Nano-SiO_2_ acts not only as an active component that accelerates cement hydration by providing additional nucleation sites for C-S-H hydrates, but also as a nanofiller that effectively reduces the porosity of the cement matrix. These processes lead to the filling of voids in the microstructure of the hardened cement paste, resulting in the densification of the microstructure, particularly in the interfacial transition zone between the aggregate and the cement matrix. Additionally, the presence of nanosilica contributes to the reduction in portlandite (Ca(OH)_2_) due to its secondary reaction with nano-SiO_2_, which promotes the formation of additional hydration products, primarily C-S-H gel. Improved microstructural densification in the interfacial transition zone enhances the cohesion and homogeneity of the material, reducing the quantity and size of defects. Consequently, the mechanical properties and durability of nano-SiO_2_ modified mortars are significantly improved. The obtained results are consistent with the SEM observations presented in Figure 5, which further confirm the enhanced microstructural densification of the nanosilica-modified mortars.

The obtained compressive strength results show clear consistency with the trends reported in the literature. In the tested mortars, a systematic increase in compressive strength was observed with increasing nano-SiO_2_ content up to 2.914% of the binder mass, which corresponds to the range considered optimal by many authors. The vast majority of studies indicate that nano-SiO_2_ dosages within the range of 2–5% lead to the greatest improvement in mechanical properties, primarily due to microstructure densification and intensified hydration of the C_3_S and C_2_S phases. The literature emphasizes that a 3% addition of nano-SiO_2_ can increase the compressive strength of cement pastes by up to 30% compared to control samples, particularly at early curing ages. The findings of Horszczaruk [36] and other researchers [9,10,12] confirm that a dosage of approximately 3% is especially effective due to the high pozzolanic reactivity of nano-SiO_2_ and its pore-filling effect. A similar trend was observed in the present study-the increase in compressive strength fell within the range reported in the literature, which clearly confirms the effectiveness of the applied nanomodification.

The results obtained regarding fracture resistance are consistent with the trends reported in the literature, although the range of nano-SiO_2_ dosages analysed here differs from those used by other researchers. In this study, a clear increase in fracture toughness *K_Ic_^S^* was observed with increasing nano-SiO_2_ content up to 2.914%, combined with a reduction in the water-to-binder ratio. This tendency corresponds to the findings of the authors in [18], who reported that fracture energy increases with the addition of colloidal nanosilica up to approximately 3%, followed by a decrease at higher dosages. A similar pattern was identified by Zhang et al. [19], who observed an improvement in fracture parameters up to about 5% nano-SiO_2_, and a subsequent decline with further increases in dosage. Importantly, the “favourable threshold dosage” of nanosilica frequently described in the literature is fully consistent with the logic of the results obtained in this work: at low and moderate nanosilica contents, microstructural densification leads to enhanced fracture resistance, whereas at higher additions (above 3–5%) agglomeration effects dominate, resulting in a deterioration of mechanical performance. In this study, the maximum nano-SiO_2_ dosage of 2.914% falls within the range identified as optimal, which explains the continued increase in fracture resistance observed in the tested mortars.

**Figure 6:**
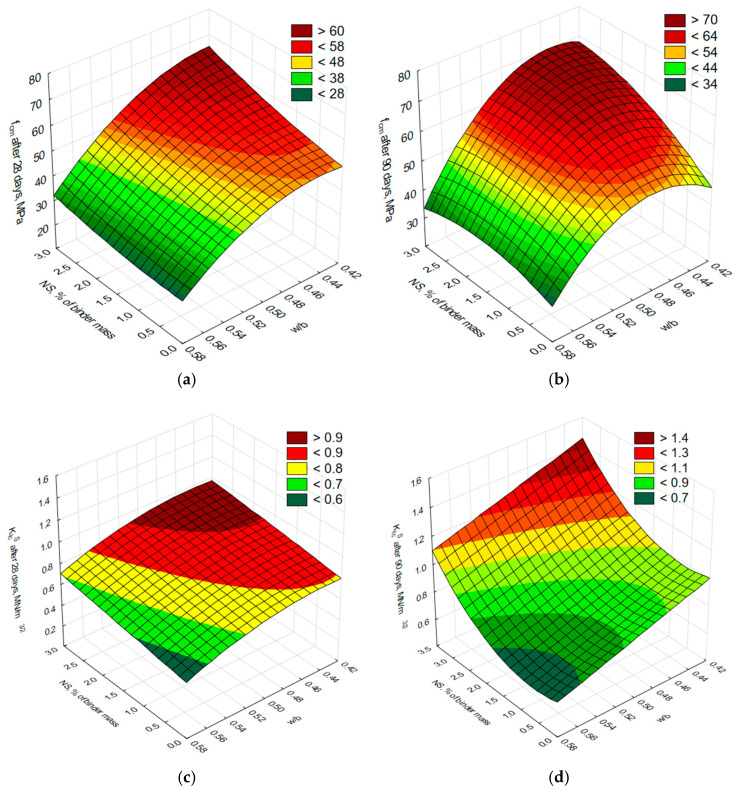
Surface plots showing the relationships between compressive strength (*f_cm_*) and fracture toughness expressed by the critical stress intensity factor (*K_Ic_^S^*) and the water-to-binder ratio and nanosilica content after 28 days ((**a**) compressive strength, (**c**) fracture toughness expressed by the critical stress intensity factor) and 90 days ((**b**) compressive strength, (**d**) fracture toughness expressed by the critical stress intensity factor) of curing for nano-SiO_2_ modified mortars.

To determine the influence of varying nanosilica content and the water-to-binder ratio on compressive strength and fracture resistance, expressed by the critical stress intensity factor (*K_Ic_^S^*), the test results were subjected to statistical analysis. This analysis led to the development of a statistical model. Tests for equality of means were conducted, the homogeneity of variance was verified, the significance of effects and the coefficients of approximation equations were assessed, and the adequacy of the resulting regression function was evaluated. The results of the statistical analyses, presented in Table 9 and Table 10, confirm that the condition of variance homogeneity was met for both studied properties and demonstrate a statistically significant influence of the adopted independent variables on the examined properties. The obtained response surfaces for both properties, after eliminating statistically insignificant coefficients, are shown in Figure 6, while the functional relationships along with the correlation coefficient (*R*) for the model are presented in Table 11.

Based on the regression models for compressive strength after 28 days of curing of cement composites modified with nano-SiO_2_, it was found that at the highest water-to-binder ratios (0.55–0.57), the compressive strength of these composites did not increase or showed only a negligible increase, regardless of the amount of modifier added. A more noticeable increase in the compressive strength of nano-SiO_2_ modified cement composites was observed only at *w/b* ratios below 0.52, particularly in mortars with a lower *w/b* ratio and a higher content of the modifier. The regression model for compressive strength after 90 days indicated that the highest values were achieved at *w/b* ratios in the range of 0.43–0.5 and with a nano-SiO_2_ content of 1% to 2.9% of the binder mass. The lowest compressive strength values were recorded at a *w/b* ratio of 0.55–0.57, regardless of the nanosilica content in the composite.

For the regression model of fracture toughness measured by the critical stress intensity factor (*K_Ic_^S^*) after 28 days of curing of cement composites modified with nano-SiO_2_, it was found that the lowest *K_Ic_^S^* values were obtained at a water-to-binder ratio (*w/b*) ranging from 0.54 to approximately 0.57 and with a nano-SiO_2_ content of up to around 1%. Conversely, the highest *K_Ic_^S^* values were achieved at *w/b* ratios between 0.43 and approximately 0.48, with nano-SiO_2_ contents ranging from about 2% to 2.9%. It is important to remember that the interpretation of results is limited to the domain covered by the experiment. After 90 days of curing, a clear influence of increased nano-SiO_2_ content on the rise in *K_Ic_^S^* was observed, especially at higher content levels. The maximum *K_Ic_^S^* shifted toward cement composites modified with the highest amount of nano-SiO_2_, even at slightly higher *w/b* ratios. However, an increase in the *w/b* ratio significantly deteriorated fracture toughness measured by the *K_Ic_^S^* factor.

### 3.4. Influence of Nano-SiO_2_ Content on the Load–Deformation (CMOD) Relationship

Based on the analysis of the load–crack mouth opening displacement (*CMOD*) curves of the tested mortars, significant variations in the obtained curves were observed as a result of introducing the nano-silica modifier.

**Figure 7:**
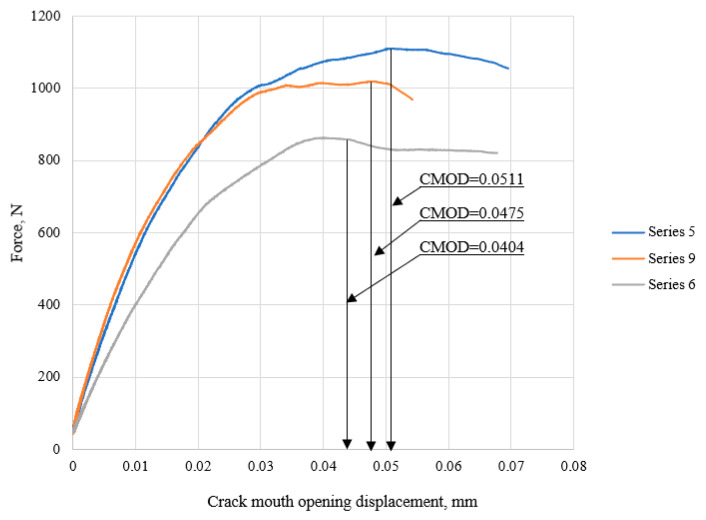
Comparison of representative *P–CMOD* fracture curves obtained for specimens made from mortars containing 1.5% nanosilica and varying *w/b* ratios of 0.429 (series 5), 0.5 (series 9) and 0.57 (series 6) after 28 days of curing.

A comparative analysis of the load–crack mouth opening displacement (*CMOD*) curves was performed for cement composites modified with the same amount of nano-silica but with varying water-to-binder *(w/b*) ratios (Figure 7). It was found that the sample with a *w/b* ratio of 0.429 (series 5) was capable of carrying a higher maximum load. The representative curves for samples with *w/b* ratios of 0.429 (series 5) and 0.5 (series 9) showed an extended range of linear load–deformation (*CMOD*) relationship, indicating a smaller increase in deformation with rising load. In the case of series 6 (*w/b* = 0.57), the curve reached the lowest maximum load, and the linear segment of the graph had a lower slope, indicating significantly lower stiffness and greater susceptibility to deformation under increasing load.

**Figure 8:**
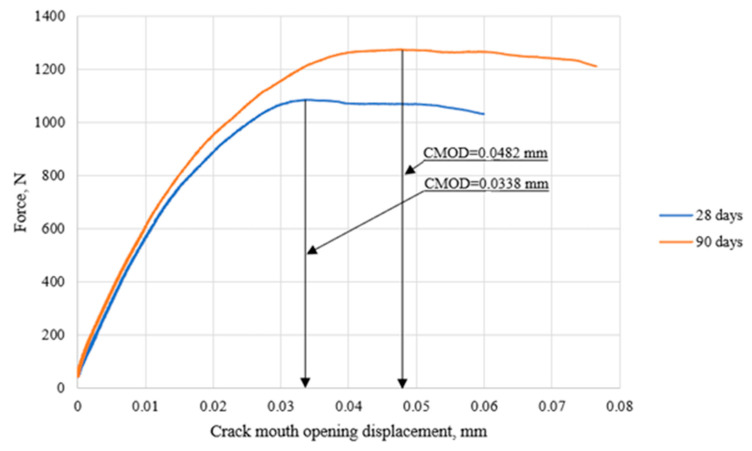
Comparison of representative *P–CMOD* fracture curves obtained for specimens made from mortar containing 2.914% nanosilica and a *w/b* ratio of 0.5 (series 7) after 28 and 90 days of curing.

In the case of cement composites modified with nano-silica at a *w/b* ratio of 0.5 and a nano-silica content of 2.914% by binder mass (series 7 mortars), an increase in the slope of the linear segment of the initial phase of the *P–CMOD* curve was observed, along with its extension as the mortar aged (Figure 8). This indicates greater material stiffness, meaning less susceptibility to deformation under the same load level. The reduction in deformation corresponds to an increase in the flexural modulus of elasticity *E*. It was found that nano-silica-modified cement composites are capable of carrying higher maximum loads as they age. The maximum load was reached at greater displacement values of the contact points of the extensometer used for measuring the crack mouth opening displacement (*CMOD*).

### 3.5. Fractal Examinations

Fractal analysis of mortar fracture surfaces was conducted on randomly selected fracture surfaces from five samples within each mortar series. Profile lines were delineated on the fracture surfaces in the direction of crack propagation. The analysis was performed on samples after 28 and 90 days of curing. A total of 24 fracture surfaces were analyzed—one from each series of modified and unmodified mortars. The results of the fractographic analysis are presented in Table 12.

The analysis of the fractal dimension *D* revealed a decrease in its value across all mortar series with curing time. In mortars unmodified with nanosilica, an increase in the fractal dimension *D* was observed with an increasing water-to-binder ratio. The lowest values of fractal dimension *D* were recorded for mortars with low *w/b* ratios ranging from 0.429 to 0.5 and with high nanosilica contents between 2.5% and 2.914%, where this decrease amounted to approximately 5% compared to the control mortars. This indicates the formation of fracture surfaces with reduced roughness during cracking, which, along with high values of the tested parameters (*f_cm_*, *K_Ic_^S^*), points to a significant improvement in the microstructure of these mortars. It was also observed that an increase in the *w/b* ratio and a decrease in nanosilica content further increased the fractal dimension *D*. More complex fracture surfaces in mortars with higher *w/b* ratios suggest the presence of more structural defects, leading to structural heterogeneity and reduced cohesion between the aggregate and the cement matrix. The results showed that at the same water to binder ratio, lower values of the fractal dimension *D* were obtained in mortars with higher nanosilica content. Mortars that exhibited flatter fracture surfaces reflected by lower *D* values also demonstrated high crack resistance and compressive strength. It should be emphasized that the fractal characteristics of fracture surfaces in concrete can be strongly influenced by the particle size distribution of the aggregate [37]. However, this effect is considerably less significant in the analysed mortars, where the maximum aggregate size does not exceed 2 mm.

The conducted studies were subjected to further statistical analysis to determine the functional relationships between the fractal dimension *D* and the independent variables used in the experimental design. For this purpose, as in the case of *f_cm_* and *K_Ic_^S^* an ANOVA analysis was performed to assess the significance of the effects. The final regression models are presented in Table 13 and in Figure 8.

**Figure 9:**
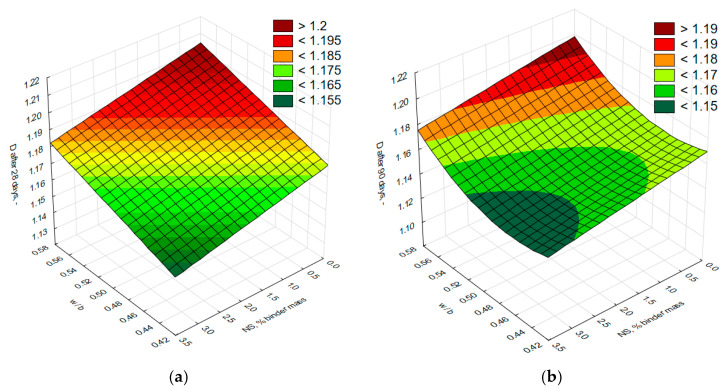
(**a**,**b**) Surface plots of the relationship between the fractal dimension *D* of the profile line and the water to binder ratio and nanosilica content after 28 and 90 days of curing.

The statistically significant effects in the regression model of the fractal dimension *D* for cement composites modified with nano-SiO_2_ after 28 days of curing were the linear main effects of both variables, i.e., the water-to-binder ratio and the nano-silica content relative to the binder mass. It was demonstrated that a decrease in the water-to-binder ratio and an increase in the nano-silica content resulted in a decrease in the fractal dimension *D* (Figure 9a). The lowest values of the fractal dimension were obtained for profile lines extracted from fracture surfaces of mortars with a *w/b* ratio below 0.45 and nano-silica content above 2.7%. The lower fractal dimension within these ranges indicates a smoother surface of the hardened cement paste compared to that of unmodified paste. The inclusion of nano-silica, with finer particle size than cement grains, led to the filling of voids between hydrated cement grains with pozzolanic reaction products from nano-silica reacting with Ca(OH)_2_. Greater surface roughness of the fracture surfaces was observed in mortars with a *w/b* ratio ranging from approximately 0.55 to 0.58 and a nano-SiO_2_ content up to 0.5% relative to the binder mass. The higher roughness of fracture surfaces in mortars with higher *w/b* ratios can be explained by increased porosity in the hydration products.

Analyzing the graph (Figure 9b) illustrating the relationship between the fractal dimension *D* of the profile line of cement composites modified with nano-silica and the independent variables after 90 days of curing, a linear relationship was observed—similar to that found in composites after 28 days—between the fractal dimension and the amount of the nano-SiO_2_ modifier. As the nano-silica content and the age of the mortar increase, the fractal dimension value decreases. This reduction results from ongoing cement hydration and pozzolanic reactions of the nano-silica, which contribute to enhanced compactness and homogeneity of the hardened mortar’s microstructure.

### 3.6. The Relationship Between the Critical Stress Intensity Factor K_Ic_^S^ and the Fractal Dimension D of Mortars Modified with Nano-SiO_2_

The statistical analysis of the test results allowed for identifying a relationship between the fractal dimension *D* of the profile line and the fracture toughness described by the critical stress intensity factor *K_Ic_^S^* of mortars modified with nano-SiO_2_. An increase in the critical stress intensity factor corresponds with a simultaneous decrease in the fractal dimension *D* of the profile line. Statistically significant linear relationships were demonstrated as follows:

▪ For modified mortars after 28 days,

(7) $$K_{Ic}^{S\;28}=12.137-9.597\cdot D^{28}$$

▪ For modified mortars after 90 days,

(8) $$K_{Ic}^{S\;90}=13.903-11.178\cdot D^{90}$$

**Figure 10:**
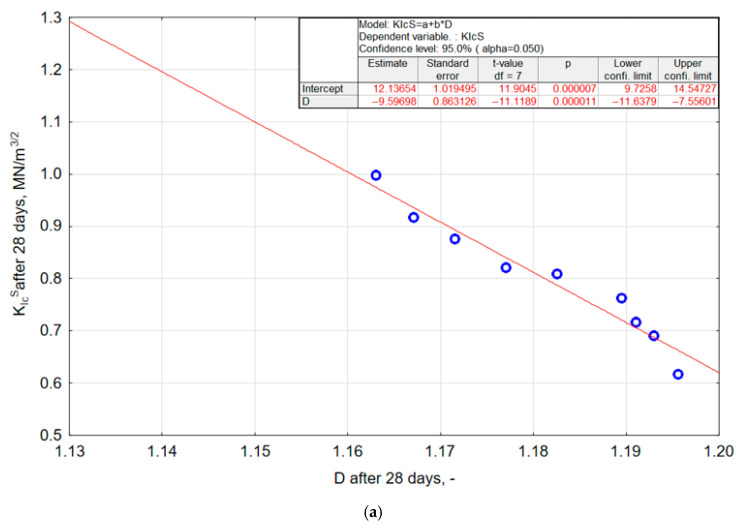

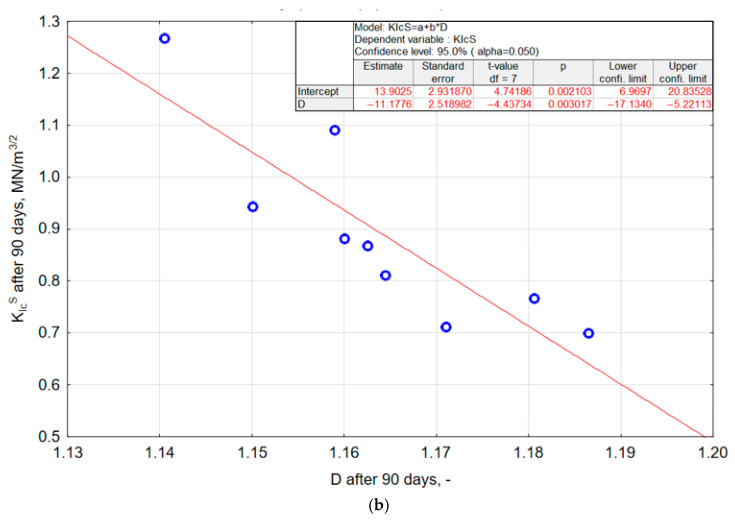
Relationship between the fractal dimension *D* of the profile line and the critical stress intensity factor *K_Ic_^S^* for nano-silica-modified mortars after 28 days (**a**) and 90 days (**b**) of curing.

The linear correlation coefficients for the models are *R* = 0.973 for model (7) and *R* = 0.859 for model (8). The coefficients of determination are *R^2^* = 0.945 for model (7) and *R^2^* = 0.738 for model (8), respectively. The obtained linear correlation coefficients *R* indicate a very strong relationship between the fractal dimension *D* and the fracture toughness *K_Ic_^S^*. The 28-day model exhibits an almost perfect correlation (*R* = 0.973), while the 90-day model still demonstrates a high level of dependence (*R* = 0.859), confirming the stability of this relationship even at a more advanced curing age. The high values of the determination coefficient (*R*^2^ = 0.945 for 28 days and *R*^2^ = 0.738 for 90 days) show that the models explain a substantial proportion of the variability in fracture toughness based on the fractal dimension *D*. For the 28-day samples, nearly 95% of the variability in *K_Ic_^S^* is captured by the linear model, whereas for the 90-day samples the model explains more than 73%, which remains a very good level of fit for composite material research. The significance analysis of the coefficients in the regression models (7) and (8) confirmed the statistical significance of both coefficients. The obtained relationships (7) and (8) are presented in Figure 10a and Figure 10b, respectively.

The use of nanosilica as a modifier had a positive effect on both the fractal dimension *D* of the profile line and the value of the critical stress intensity factor *K_Ic_^S^*. It was found that as the value of the critical stress intensity factor *K_Ic_^S^* increases, the fractal dimension *D* decreases.

The varied slope of both relationships (Figure 10) indicates the influence of mortar age on the relationship between the fractal dimension *D* and the critical stress intensity factor *K_Ic_^S^*. A greater dynamic increase in fracture resistance according to the Mode I fracture model (as indicated by the *K_Ic_^S^* value) with a decrease in the fractal dimension D was observed in 28-day mortars compared to 90 day mortars. The microstructure in 28 day nanosilica modified composites develops more intensively and dynamically, which may contribute to improved properties such as fracture resistance. During the first 28 days, significant chemical and physical processes occur that shape the material’s structure, potentially affecting its strength. After 90 days, when the microstructure reaches a more stable state, changes become less pronounced. The application of nanosilica as a modifier contributed to improvements in both the fractal dimension and fracture toughness. Both properties can be described using linear relationships. It should also be noted that the obtained models are empirical, and their applicability is limited to the variable range covered in the experiment: water-to-binder ratios of 0.43–0.57, nano-SiO_2_ content of 0.086–2.914%, and mortar ages of 28 and 90 days. Extrapolating the models beyond these boundaries may lead to inaccurate predictions, as the linear relationships describe the material behaviour only within the experimentally investigated domain.

The analysis of the relationship between the critical stress intensity factor *K_Ic_^S^* and the fractal dimension *D* demonstrated that the increase in fracture resistance of mortars modified with nano-SiO_2_ is closely correlated with the decrease in fracture surface roughness. The reduction in *D* reflects the smoothing and densification of the fracture surface resulting from decreased porosity and the limitation of structural defects in the interfacial transition zone due to pore-filling effects and the pozzolanic reaction of nano-SiO_2_. At the same time, the increase in *K_Ic_^S^* confirms the improvement in cohesion and homogeneity of the cement matrix, which hinders crack initiation and propagation. The microstructural basis of this relationship is clearly visible in the SEM images, which show a denser structure and reduced void content in nano-SiO_2_ modified mortars compared with the reference sample. These findings unequivocally confirm that nano-SiO_2_ enhances both the microstructure of the composite and its resistance to fracture.

By additionally including the variable age of the mortar in the *K_Ic_^S^*–*D* relationship, the following model was obtained:

(9) $$K_{Ic}^{S}(t)=13.237-10.501\cdot D\left(t\right)-0.001\cdot t$$

where *K_Ic_^S^(t)*—critical stress intensity factor at time *t*; *D(t)*—fractal dimension as a function of mortar age; *t*—time, days.

The correlation coefficient of *R* = 0.905 for model (9) indicates a very strong linear relationship between the predicted and observed values. The measurement points are distributed close to the trend line (*y* = *x*), which confirms the high consistency of the model predictions with the experimental data. The small deviations between the points and the ideal fit line suggest that the variability of *K_Ic_^S^* is largely explained by the model incorporating both the mortar age and the fractal dimension of the fracture surface. The significance analysis of the coefficients (*t*-test, *p* < 0.05) confirms that all model parameters—both the intercept and the coefficients associated with *D(t)* and *t*—are statistically significant.

**Figure 11:**
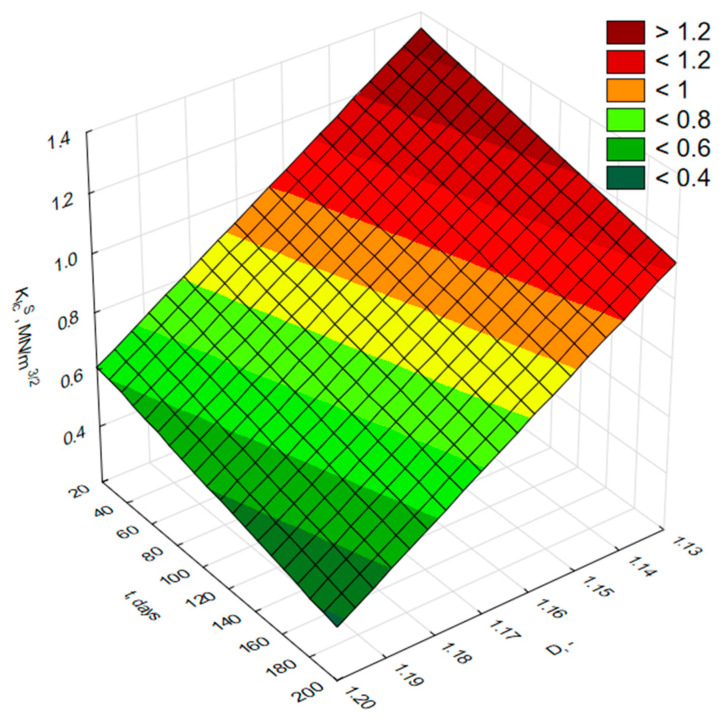
Surface plot of the *K_Ic_^S^(t)—D(t)* relationship after t days of curing for modified mortars.

**Figure 12:**
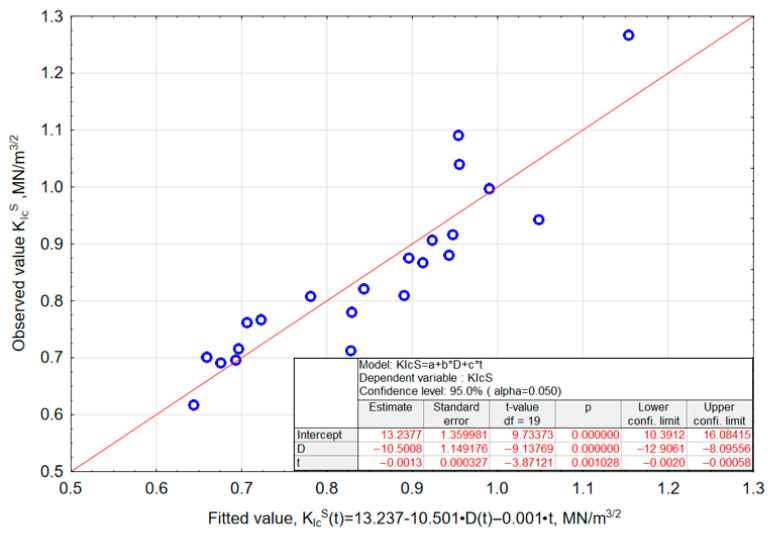
Plot of observed vs. predicted values with results of the significance analysis of model (9) coefficients.

The surface plot illustrating the relationship between *K_Ic_^S^(t)* and D(t), taking into account the age of the modified mortar, is presented in Figure 11, while the relationship between observed and predicted values calculated from model (9) is shown in Figure 12.

**Figure 13:**
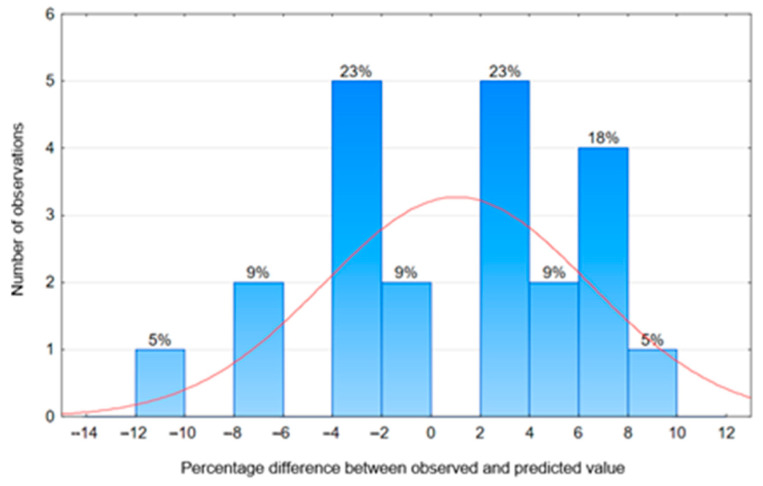
Histogram of percentage differences between observed and predicted values of the critical stress intensity factor *K_Ic_^S^* for nano-silica-modified mortars according to model (9).

The analysis of the histogram showing the percentage differences between the observed and predicted values of the critical stress intensity factor *K_Ic_^S^* for nano-silica-modified mortars, presented in Figure 13, confirms the reliability of the proposed model (9). Most data fall within the range of −4% to +4%, indicating high prediction accuracy and minimal deviations from actual values. The extreme percentage differences between the observed and predicted *K_Ic_^S^* values based on model (9) ranged from −12% to +10%, with 90% of the cases not exceeding 10%. The red curve represents the fitted normal distribution, which allows for assessing the conformity of the percentage differences between the observed and predicted values with the theoretical distribution. The nature of the obtained error distribution suggests that model (9) can be effectively used to predict the fracture toughness according to the first model of nano-SiO_2_ modified cement composites, potentially reducing the need for costly and time-consuming experimental testing.

## 4. Conclusions

Based on the conducted research, the following conclusions were drawn:

Mortars modified with nano-silica, used as a partial replacement for cement in amounts up to approximately 2.914% of the cement mass, exhibit higher compressive strength and fracture resistance compared to mortars not modified with nano-silica, both after 28 and 90 days of curing. The maximum increase in compressive strength for nano-SiO_2_ modified mortars reached 68%, while the maximum increase in fracture toughness expressed by *K_Ic_^S^* was 63%. The improvement in both fracture resistance and compressive strength in nano-SiO_2_ modified mortars results not only from the reduction in the water to binder ratio but also from the increased proportion of nano-silica relative to the binder mass. The results clearly indicate that an appropriately selected combination of compositional parameters—particularly the *w/b* ratio and the nano-SiO_2_ content—constitutes a key factor governing the improvement of the mechanical properties of the cement composite.

The fractal dimension of profile lines, representing a quantitative measure of fracture surface roughness resulting from material failure, is significantly influenced by the nanosilica content in the binder and the water-to-binder (*w/b*) ratio. An increase in the nanosilica content relative to the binder mass, combined with a decrease in the water-to-binder ratio, results in a lower fractal dimension. It was also found that the fractal dimension *D* decreases with the aging of the nano-SiO_2_ modified cement composite. The decrease in the fractal dimension *D* indicates enhanced homogeneity and pronounced densification of the composite’s microstructure, which leads to the formation of fracture surfaces with a lower degree of geometric complexity.

Based on the analysis of the load–deformation (*CMOD*) curves of the tested composites, it was found that the introduction of the nano-SiO_2_ modifier resulted in variations in the obtained curves. Cement composites with a higher nanosilica content and a lower water to binder ratio exhibited a steeper slope in the initial phase of the *P–CMOD* curve and an extended duration of this phase during curing, indicating increased stiffness and reduced deformability of the material. The observed increase in maximum load and the corresponding displacement at peak load confirm that nanosilica-modified composites gain strength over time.

The obtained test results demonstrate a strong correlation between the properties of the tested mortars modified with varying amounts of nano-SiO_2_ and the microstructure of the composite, described by the fractal dimension, which characterizes the fracture surfaces formed as a result of failure. As the fracture toughness of nano-SiO_2_ modified cement composites increases, the profile lines extracted from the fracture surfaces become flatter and exhibit a lower fractal dimension. This indicates an increase in the homogeneity of these composites compared to those unmodified with nano-SiO_2_. A statistically significant relationship was demonstrated between fracture toughness, expressed by the critical stress intensity factor *K_Ic_^S^*, and the fractal dimension *D* in mortars modified with varying nanosilica content. The predicted values determined using the developed predictive models show strong agreement with experimental data, as confirmed by the high value and statistical significance of the correlation coefficients *R*. The resulting regression models can be used in the design of nano-SiO_2_ modified composites to achieve desired properties.

It should be emphasized that the obtained results apply only to the range of variables adopted in the experimental design, i.e., the *w/b* ratio of 0.43–0.57, the nano-SiO_2_ content of 0.086–2.914% of the binder mass, and the two curing periods analyzed (28 and 90 days). In the tested systems, nanosilica was present partly in the form of agglomerates, which may have affected the effective reactivity of the modifier and the resulting mechanical properties. Future research should include expanding the experimental scope to a wider range of nano-SiO_2_ dosages, different dispersion methods, alternative types of cement and aggregates, as well as additional microstructural characterization techniques and validation of the proposed predictive models on other cementitious composites, allowing for a more comprehensive assessment of their universality and practical applicability.

## Figures and Tables

**Table 1:** Chemical and mineralogical composition of “Ożarów S.A.” cement.

Component Name	Component Designation	Content, % by Mass
Calcium oxide	CaO	64.49
Silicon oxide	SiO_2_	20.32
Aluminum oxide	Al_2_O_3_	5.28
Iron(III) oxide	Fe_2_O_3_	4.05
Iron(II) oxide	FeO	-
Magnesium oxide	MgO	0.74
Chloride ion content	Cl	0.04
Insoluble residue	Pn	0.93
Loss on ignition	Sp	2.74
Saturation coefficient	Wn	0.94
Silica modulus	n	2.26
Alumina modulus	p	1.31
Tricalcium silicate	C_3_S	64.6
Dicalcium silicate	C_2_S	12.6
Tricalcium aluminate	C_3_A	9.4
Tetracalcium aluminoferrite	C_4_AF	6.9

**Table 2:** Properties of nanosilica (nano-SiO_2_).

Property	Values
Appearance	White powder
Particle size, nm	10–15
SSA, m^2^/g	200–300
Purity, %	≥99.8
PH value	5–7
Bulk density, g/cm^3^	0.15

**Table 3:** Summary of component quantities for modified and unmodified mortars per 1 m^3^.

Series	*w*/*b*	*NS*, % of Binder Mass	Component Quantities			
			Cement, g	Nano-SiO_2_, g	Water, g	Sand, g
1	0.45	2.5	571.3	14.6	263.7	1757.8
2	0.45	0.5	583.0	2.9	263.7	
3	0.55	2.5	571.3	14.6	322.3	
4	0.55	0.5	583.0	2.9	322.3	
5	0.429	1.5	577.1	8.8	247.6	
6	0.57	1.5	577.1	8.8	333.9	
7	0.5	2.914	568.8	17.1	292.9	
8	0.5	0.086	585.4	0.5	292.9	
9	0.5	1.5	577.1	8.79	865.4	
A	0.45	-	585.9	-	263,7	
B	0.5	-	585.9	-	865.4	
C	0.55	-	585.9	-	322.3	

**Table 4:** Preliminary test results of compressive strength for series 7 mortar (*w/b* = 0.5, *NS* = 2.9%) and series 3 mortar (*w/b* = 0.55, *NS* = 0.5%) after 3 and 7 days of curing.

*w/b*	*NS*, % of Binder Mass	Compressive Strength *f_cm_*, MPa					
		Age, Days	Mean	Min.	Max.	Standard Deviation	Standard Error of the Mean
0.5	2.9	3	29.04	20.79	37.62	5.67	1.89
		7	51.59	46.21	59.84	7.25	4.18
0.55	0.5	3	20.54	12.18	17.33	3.45	1.42
		7	30.34	28.66	32.33	1.85	1.07

**Table 5:** Sonication time depending on nano-SiO_2_ content relative to binder mass for compressive strength test specimens.

Nano-SiO_2_ Content, % of Binder Mass	Ultrasonic Sonication Time, Min
0.086	3
0.5	10
1.5	15
2.5	20
2.914	22

**Table 6:** Ultrasonic sonication time depending on nano-SiO_2_ content relative to binder mass for fracture resistance test specimens according to Mode I fracture toughness.

Nano-SiO_2_ Content, % of Binder Mass	Ultrasonic Sonication Time, Min
0.086	5
0.5	15
1.5	25
2.5	40
2.914	45

**Table 7:** Workability test results of nano-SiO_2_ modified mortars and reference mortars without nano-SiO_2_.

Mortar Series	*w/b*	*NS*, % of Binder Mass	Superplasticizer, % of Binder Mass	Flow, mm	
				with Nano-SiO_2_	Without Nano-SiO_2_
1	0.45	2.5	3.00	160.0	-
2	0.45	0.5	3.00	150.8	-
3	0.55	2.5	1.11	150.8	-
4	0.55	0.5	0.44	160.0	-
5	0.429	1.5	3.00	160.0	-
6	0.57	1.5	0.67	160.0	-
7	0.5	2.914	3.00	150.8	-
8	0.5	0.086	1.11	160.0	-
9	0.5	1.5	3.00	150.5	-
A	0.45	-	1.11	-	150.8
B	0.5	-	0.67	-	150.5
C	0.55	-	0.00	-	150.5

**Table 8:** Test results of compressive strength (*f_cm_*) and critical stress intensity factor (*K_Ic_^S^*) after 28 and 90 days of curing for nano-SiO_2_ modified cement composites (1–9) and unmodified cement composites (series A, B, C).

Series Mortar	*w/b*	*NS*, % of Binder Mass	Mortar Properties			
			After 28 Days of Curing		After 90 Days of Curing	
			*f_cm_* ± Standard Error of the Mean, MPa	*K_Ic_^S^* ± Standard Error of the Mean, MN/m^3/2^	*f_cm_* ± Standard Error of the Mean, MPa	*K_Ic_^S^* ± Standard Error of the Mean, MN/m^3/2^
1	0.45	2.5	60.0 ± 0.24	0.877 ± 0.018	67.2 ± 0.34	0.943 ± 0.028
2	0.45	0.5	51.4 ± 0.22	0.810 ± 0.026	54.9 ± 0.20	0.868 ± 0.013
3	0.55	2.5	38.9 ± 0.30	0.692 ± 0.019	46.9 ± 0.21	0.767 ± 0.035
4	0.55	0.5	34.8 ± 0.23	0.618 ± 0.025	43.4 ± 0.19	0.701 ± 0.048
5	0.429	1.5	56.2 ± 0.30	0.917 ± 0.023	65.0 ± 0.26	1.091 ± 0.050
6	0.57	1.5	33.9 ± 0.27	0.717 ± 0.031	45.2 ± 0.26	0.713 ± 0.030
7	0.5	2.914	57.7 ± 0.18	0.998 ± 0.028	67.3 ± 0.21	1.267 ± 0.067
8	0.5	0.086	46.8 ± 0.11	0.763 ± 0.025	56.1 ± 0.35	0.811 ± 0.028
9	0.5	1.5	49.8 ± 0.26	0.821 ± 0.029	56.1 ± 0.35	0.882 ± 0.026
A	0.45	-	42.9 ± 0.28	0.799 ± 0.016	51.8 ± 0.33	0.807 ± 0.093
B	0.5	-	37.2 ± 0.52	0.677 ± 0.045	47.6 ± 0.12	0.773 ± 0.038
C	0.55	-	30.4 ± 0.35	0.511 ± 0.041	43.0 ± 0.24	0.566 ± 0.020

**Table 9:** Results of statistical analysis of variance homogeneity for compressive strength and fracture toughness expressed by the critical stress intensity factor *K_Ic_^S^* of nano-SiO_2_ modified mortars after 28 and 90 days of curing.

	Significance Level *p*	
	After 28 Days of Curing	After 90 Days of Curing
Compressive strength, *f_cm_*	0.19	0.25
Critical stress intensity factor, *K_Ic_^S^*	0.19	0.06

**Table 10:** Results of statistical analysis of mean equality for compressive strength and fracture toughness expressed by the critical stress intensity factor *K_Ic_^S^* of nano-SiO_2_ modified mortars after 28 and 90 days of curing.

	Significance Level *p*	
	After 28 Days of Curing	After 90 Days of Curing
Compressive strength, *f_cm_*	close to 0	close to 0
Critical stress intensity factor, *K_Ic_^S^*	close to 0	close to 0

**Table 11:** Developed regression models for compressive strength (*f_cm_*) and fracture toughness expressed by the critical stress intensity factor (*K_Ic_^S^*) of nano-SiO_2_ modified mortars after 28 and 90 days of curing, along with correlation coefficients (*R*).

Age, Day	Properties, Model		*R*	*N*
28	$f_{cm}=49.84+7.01\cdot NS+1.24\cdot {\left(NS\right)}^{2}-17.41\cdot w/b-6.09\cdot {\left(w/b\right)}^{2}-2.26\cdot NS\cdot b$	(1)	0.987	54
	$K_{Ic}^{S}=0.829+0.118\cdot NS-0.166\cdot w/s-0.062\cdot {\left(w/b\right)}^{2}$	(2)	0.802	45
90	$f_{cm}=63.07+7.96\cdot NS-3.94\cdot {\left(NS\right)}^{2}-15.09\cdot b-10.74\cdot {\left(w/b\right)}^{2}-4.38\cdot NS\cdot w/b$	(3)	0.960	54
	$K_{Ic}^{S}=0.841+0.196\cdot NS+0.118\cdot {\left(NS\right)}^{2}-0.220\cdot w/b$	(4)	0.760	45

where *w/b*—water-to-binder ratio; *NS*—percentage of nanosilica relative to binder mass; *R*—correlation coefficient given for all results; *N*—number of results.

**Table 12:** Fractal dimension *D* of profile lines extracted from fracture surfaces of nano-silica-modified and unmodified mortars.

Mortar Series	*w/b*	*NS*, % of Binder Mass	Fractal Dimension *D*, ± Standard Error of the Mean, -	
			28 days	90 Days
			*D*	*D*
1	0.45	2.5	1.172 ± 0.003	1.150 ± 0.004
2	0.45	0.5	1.183 ± 0.004	1.163 ± 0.002
3	0.55	2.5	1.193 ± 0.003	1.181 ± 0.005
4	0.55	0.5	1.196 ± 0.004	1.187 ± 0.003
5	0.429	1.5	1.167 ± 0.004	1.159 ± 0.010
6	0.57	1.5	1.191 ± 0.005	1.171 ± 0.003
7	0.5	2.914	1.163 ± 0.003	1.140 ± 0.003
8	0.5	0.086	1.190 ± 0.005	1.165 ± 0.003
9	0.5	1.5	1.177 ± 0.005	1.160 ± 0.006
A	0.45	-	1.233 ± 0.003	1.224 ± 0.004
B	0.5	-	1.224 ± 0.002	1.212 ± 0.005
C	0.55	-	1.241 ± 0.003	1.240 ± 0.003

**Table 13:** Regression models for the fractal dimension *D* of profile lines extracted from fracture surfaces of nano-SiO_2_ modified mortars.

Age, Days	Fractal Dimension *D*, Model		*R*	*N*
28	D=1.181−0.017·w/b−0.013·NS	(5)	0.882	180
90	$D=1.158+0.018\cdot w/b-0.012\cdot {w/b}^{2}+0.013\cdot NS$	(6)	0.847	180

where *w/b*—water to binder ratio; *NS*—nanosilica content relative to the binder mass; *R*—correlation coefficient given for mean values; *N*—number of results.

## Data Availability

The original contributions presented in this study are included in the article. Further inquiries can be directed to the corresponding author.
